# Cost-Effectiveness of GaitSmart and an Artificial Intelligence Solution for Rehabilitation of Patients Undergoing Total Hip Arthroplasty (THA) and Total Knee Arthroplasty (TKA) in Older Population in the United Kingdom

**DOI:** 10.3390/geriatrics9050129

**Published:** 2024-10-05

**Authors:** Fernando Zanghelini, Aisling Ponzo, Georgios Xydopoulos, Richard Fordham, Saval Khanal

**Affiliations:** Health Economics Consulting, Norwich Medical School, University of East Anglia, Norwich NR4 7UQ, UK; fernandozanghelini@gmail.com (F.Z.); a.ponzo@uea.ac.uk (A.P.); g.xydopoulos@uea.ac.uk (G.X.); r.fordham@uea.ac.uk (R.F.)

**Keywords:** GaitSmart, cost-effectiveness analysis, rehabilitation, total hip arthroplasty, total knee arthroplasty

## Abstract

Background: GaitSmart (GS) is a sensor-based digital medical device that can be used with the integrated app vGym to provide a personalised rehabilitation programme for older people undergoing total hip arthroplasty (THA) or total knee arthroplasty (TKA). This study aimed to determine whether the GS intervention used in the rehabilitation of older people undergoing THA or TKA is potentially cost-effective compared to the current standard of care (SoC). Methods: Decision-analytic modelling was conducted to estimate the cost-effectiveness over a seventeen-week time horizon from an NHS perspective. UK clinical and cost data from the GaitSmart randomised clinical trial was used to obtain the input parameters, and a sensitivity analysis was performed to address uncertainties. Results: Over a seventeen-week time horizon, GS incurred cost savings of GBP 450.56 and a 0.02 gain in quality-adjusted life years (QALYs) compared to the SoC. These results indicate that GS is the dominant intervention because the device demonstrated greater effectiveness and lower costs. Probabilistic sensitivity analyses confirm the robustness of our results. Conclusions: GS appears to offer short-term efficiency benefits and demonstrates cost-effectiveness for the improvement in gait in people undergoing THA or TKA, compared to the SoC.

## 1. Background

Osteoarthritis (OA) is a painful and debilitating condition and is the most common musculoskeletal condition in older adults [1]. According to Arthritis Research UK, in England, approximately one in five adults over 45 years have OA of the knee and one in nine have OA of the hip [2], while another study claimed that the overall prevalence of OA of the hip and knee was higher (knee: 2.9%, 95% CI: 2.7 to 2.9 and hip: 1.5%, 95% CI: 1.4 to 1.5) [3].

Furthermore, OA places a strain on scarce resources. In the UK, the total healthcare cost of OA is estimated to be over GBP 1 billion [4]. Based on an observational retrospective study designed to estimate the resource utilisation and costs in the United Kingdom (UK) patients with moderate to severe OA pain, the direct cost of healthcare at 12 months was GBP 2519 in patients with moderate to severe OA pain and GBP 3389 for those with severe OA pain [5].

Whilst treatment and management of OA involve a multidisciplinary approach and various management options, including patient education, self-management, and pharmacological and non-pharmacological treatments [6], individuals diagnosed with this condition frequently progress to needing a joint arthroplasty.

There is strong published evidence showing that total hip arthroplasty (THA) and total knee arthroplasty (TKA) are highly effective in reducing pain symptoms and functional limitations in most patients [7,8], in addition to being cost-effective [9,10]. Over 100,000 THA and TKA operations are carried out each year in the UK [11]. These numbers are projected to increase with both an increase in national life expectancy and in the prevalence of obesity, adding even further pressure on the English National Health Service’s (NHS) funding and capacity [12]. Additionally, 10% of patients who have received a THA or TKA have gait abnormalities one year post-operation [13].

This is a result of a lack of objective assessment data of post-operation gait, leaving patients to continue the walking pattern they had adopted before the operation to avoid pain in the arthritic joint, thereby creating abnormalities in their gait post-operation. Patients need to retrain their walking by strengthening weakened muscles, as by continuing the incorrect walking pattern, many continue to strain their joints and use muscles incorrectly [14]. This can lead to falls, reduced activity, or pain in other joints.

GS is a digital medical device (CE-marked class 1m medical device) that uses patient-connected motion sensors and an artificial intelligence algorithm to provide objective gait data from which personalised rehabilitation can be effectively delivered and monitored. This study aimed to conduct an economic evaluation to determine the cost-effectiveness of the GaitSmart (GS) system compared with the current standard of care (SoC) in people undergoing THA or TKA with gait and mobility issues.

Total hip arthroplasty (THA) and total knee arthroplasty (TKA) are common surgical procedures aimed at relieving pain and improving function in patients with severe hip and knee osteoarthritis [15]. Post-operative rehabilitation is crucial for optimising patient outcomes [16]. Various rehabilitation strategies exist, with GaitSmart (GS) being a novel approach that leverages technology to enhance recovery [17].

Cost-effectiveness analysis (CEA) is a method used to compare the relative costs and outcomes (effects) of different courses of action. In healthcare, CEA helps in evaluating the economic value of interventions, ensuring that resources are used efficiently to achieve the best possible health outcomes. Previous studies have demonstrated the utility of CEA in assessing rehabilitation programmes, providing a framework for evaluating both clinical and economic impacts [18,19,20]. Despite the recognised importance of CEA, there is a paucity of research specifically evaluating the cost-effectiveness of technological rehabilitation interventions such as GS compared to the standard of care (SoC). This study aimed to fill this gap by assessing the cost-effectiveness of GS in the post-operative rehabilitation of patients undergoing THA and TKA. This study aimed to conduct an economic evaluation to determine the cost-effectiveness of the GS system compared with the current SoC in people undergoing THA or TKA with gait and mobility issues.

## 2. Methods

### 2.1. Study Design

We estimated the cost-effectiveness of the GS system compared with the current SoC in people undergoing THA or TKA with gait and mobility issues in the UK. The main aspects of the analysis were summarised according to the *Consolidated Health Economic Evaluation Reporting Standards* (CHEERS) 2022 statement to increase the transparency of the proposed study [21].

### 2.2. Within-Trial Analysis

The pilot randomised controlled trial (RCT) was funded by Innovate UK under the digital health programme, whose role was to ensure the project was correctly managed. This pilot study was designed as a parallel group RCT to assess how a fully automated personalised exercise intervention (using GS) compared to standard physiotherapy (SoC) as defined in the National Institute for Health Care and Excellence (NICE) Guideline QS206 [22]. Patients were recruited from Norfolk and Norwich University Hospital (NNUH) and approved by the institutional ethics committee. All subjects had undergone hip or knee replacement using standard implants and surgical techniques. Furthermore, all patients received routine inpatient physiotherapy until discharged and then up to 6–8 weeks post-operatively. Patients were screened for eligibility criteria at 6–8 weeks post-operatively. The primary outcome measure was the change in the patient’s gait speed. This is a universally recognised measure, with established values for a mean detectable change (MDC) of 0.05 m/s and a minimally clinically significant change of 0.1 m/s [23].

We conducted an economic evaluation using data from the pilot RCT which was provided by the manufacturer (Dynamic Metrics Limited, United Kingdom). In addition to that, we have also used summary statistic data published from the same trial in this model. The clinical outcome at the start of the trial (17 weeks) was measured by the change in the patient’s gait speed. Health utilities were used to calculate the quality-adjusted life-years (QALYs) over the clinical trial period. The resource use estimates collected during the trial included physiotherapy, follow-up costs, and the costs of treatments with GS and the best SoC. Unit costs were obtained from the Personal Social Services Research Unit (PSSRU) [24] and applied to the resource use. All the costs were inflated to 2023 GBP for the analyses.

### 2.3. Study Population

The study population comprised participants who were approached at their pre-operative appointment by a member of the research team and consented to participate in the GS RCT. The target population was adults who met the following criteria: patients aged 18 years or older and undergoing THA or TKA with gait and mobility issues.

This study managed to recruit 44 participants, with 22 allocated to the intervention group (average age: 67.4 years, male proportion: 50%, average BMI: 31.05) and 22 allocated to the SoC group (average age: 72.3 years, male proportion: 50%, average BMI: 29.08). There were no discernible baseline differences between the groups [25].

### 2.4. Study Perspective

From the NHS perspective, the economic model estimated the relative cost-effectiveness of GS compared with the SoC for improving gait and mobility issues in people undergoing THA or TKA, adhering to good practice guidelines [21] and the NICE reference case [26]. Therefore, only healthcare costs (direct medical costs) related to the disease were included.

### 2.5. Intervention and Comparator

The GS is a sensor-based digital medical device (CE-marked class 1m medical device). Using an algorithm, the GS provides a detailed and objective measure of a patient’s walking ability, in which the collected data are used to automatically define a personalised exercise program. Patients assigned to the intervention group (GS) were monitored four times during the implementation of the intervention, three weeks apart. All exercises were recommended either in the Otago Exercise Program (OEP) or in the NHS older people guidance, as per the current appropriate practice. All interventions were delivered by the research team. Training of the research team was undertaken by Dynamic Metrics. To deliver the intervention, a 20-m quiet (discrete) straight corridor was used, and the patients wore flat or low-heeled shoes with proper support. The patients were instructed to use the same footwear at each appointment when possible.

The patients in the SoC group received post-operative rehabilitation according to the NICE quality standard (QS 206) [22]. All patients were advised on self-directed rehabilitation. Those who had difficulties managing activities of daily living with an ongoing functional impairment or felt that they were not achieving their goals through self-directed rehabilitation were offered group or individual outpatient rehabilitation.

Cost-effectiveness was expressed as the incremental cost-effectiveness ratio (ICER). A Monte Carlo simulation was used to calculate the 95% confidence intervals for the estimated difference in the mean cost and QALYs between the intervention groups.

### 2.6. Time Horizon

As the benefit of the GS system is usually seen over a short term, our analysis was performed over a seventeen-week time horizon—alongside the pilot GS RCT to estimate the cost-effectiveness results. This time horizon was also chosen because it is similar to the time frame in which patients receive physiotherapy (SoC) in current practice after THA and TKA. Given that our analytic time horizon was lower than 12 months, a discount rate was not applied to the costs and benefits.

### 2.7. Decision Modelling

**Figure 1:**
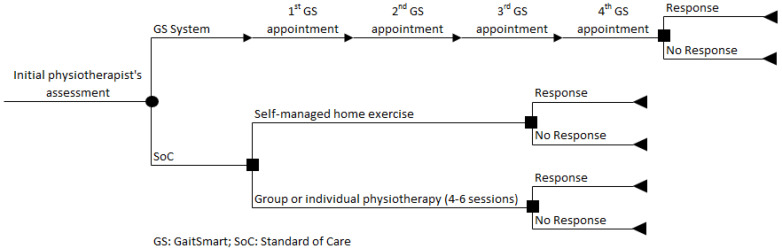
Decision tree model of GaitSmart.

The economic model is informed by a previously published early economic model [17] and is in line with the current clinical pathway described for patients undergoing THA or TKA who are eligible for SoC, according to the guidance set out by NICE (Figure 1). The seventeen weeks (GS RCT period) are represented by a decision tree model developed in Microsoft Excel 2013. In summary, patients assigned to the GS group were monitored four times, three weeks apart, while patients assigned to the SoC group were not monitored with GS and could be allocated to receive either self-managed home exercise or group or individual physiotherapy (4–6 sessions). At the end of the path, each branch of the decision tree provides the outcomes of the model (response or no response).

The decision tree modelling approach was considered appropriate as, according to expert opinion, there were no recurrent physiotherapy sessions if the patients did not respond to the initial sessions after the operation.

### 2.8. Model Input Parameters

#### 2.8.1. Transition Probabilities

The transition probabilities for patients in the SoC group who received either self-managed home exercises or group/individual physiotherapy sessions were informed by expert opinions and from the GS RCT study [25]. These probabilities were converted into appropriate parameters for our model, resulting in values of 0.2 and 0.8, respectively. Table 1, Table 2 and Table 3 present the lists of parameters used in the self-managed rehabilitation, group/individual rehabilitation, and GaitSmart model respectively.

#### 2.8.2. Costs

For the intervention cohort, we considered four twenty-minute GS sessions with a healthcare assistant and the relevant administration costs. For the SoC cohort, we considered four self-managed home sessions with a twenty-minute revision at the completion of the sessions cycle from a band 4 physiotherapist and six sessions for the group/individual physiotherapy cohort with a band 6 physiotherapist and a thirty-minute assessment with a surgical consultant at the completion of all sessions. We have also included the relevant administration costs. All medical resources costs were gathered from the *Unit Costs of Health & Social Care* 2020 report and expressed in 2023 British pounds [20].

#### 2.8.3. Health Outcomes—Clinical Effectiveness

Quality-adjusted life years (QALYs) were designated as the primary health outcome for the cost-effectiveness analysis. Health utility estimates were obtained from the GS RCT study using the EuroQol five dimension (EQ-5D 5L) questionnaire. Table 1 provides an overview of the values used in the economic evaluation, including transition probabilities, resource use, utility values, and cost measures.

#### 2.8.4. Assumptions

In the model, it is assumed that the self-managed and group-managed rehabilitation will have similar responses and effectiveness as those identified by the GS study and measured by the EQ-5D questionnaire due to lack of evidence.

#### 2.8.5. Sensitivity Analysis

To account for uncertainty in the parameter estimates used in our model, we conducted probabilistic sensitivity analyses (PSA). Instead of relying on point estimates, the parameters were defined using probability distributions. The model was then run 1000 times, each time drawing a random value from the assigned distributions. This process generated a distribution of model outputs, which were visually represented on the cost-effectiveness plane. Cost-effectiveness acceptability curves (CEACs) were used to illustrate the probability that an intervention would be cost-effective compared to the control group across various willingness-to-pay thresholds.

## 3. Results

### 3.1. Cost-Effectiveness for the Base Case

The cost-effectiveness results found that GS, compared to the SoC, had a total cost of GBP 67.00, compared to the SoC’s total cost of GBP 517.56 (f). Over a seventeen-week time horizon, the QALYs gained in the intervention group were 0.28, compared with 0.26 in the control group. This equates to a cost savings of GBP 450.56 and an incremental 0.02 QALYs gained from the use of GS. Therefore, the GS system is the dominant strategy because it is shown to be more effective and less costly compared to the SoC for improving gait and mobility issues in people undergoing THA or TKA (Table 4).

### 3.2. Probabilistic Sensitivity Analyses

**Figure 2:**
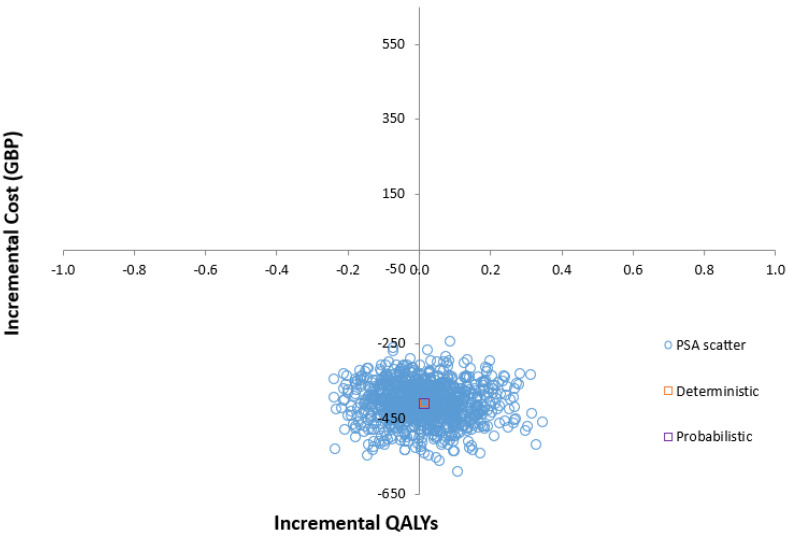
Scatter plot of incremental cost-effectiveness ratio of GS compared with SoC.

The cost-effectiveness plane (Figure 2) illustrates the results of running the model 1000 times, capturing the differences in costs and effectiveness between the GS and SoC groups. Through 1000 Monte Carlo simulations, the PSA demonstrated that at a willingness-to-pay (WTP) threshold of GBP 20,000, the GS system is dominant over the SoC in enhancing gait and mobility for individuals undergoing THA and TKA (Supplementary Table S1). While most data points fall within the southeast quadrant of the plane—indicating a ‘less costly and more effective’ dominant strategy—significant uncertainty remains regarding the magnitude and existence of the additional expected costs and QALYs.

**Figure 3:**
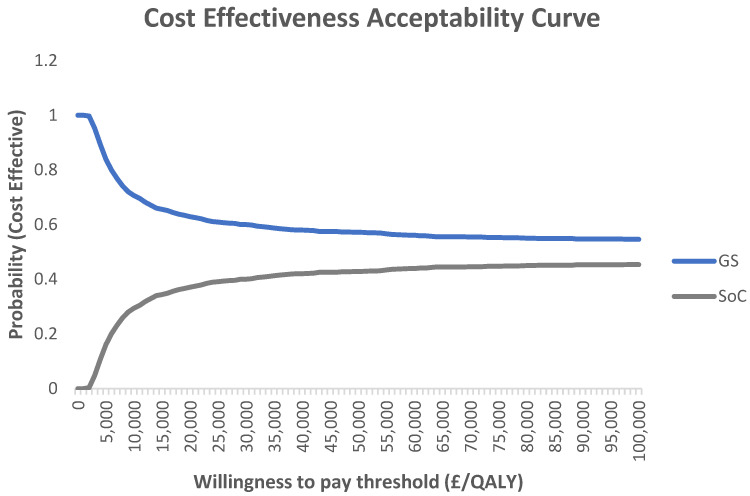
Cost-effectiveness acceptability curve (CEAC) for GS compared with SoC.

The CEAC illustrates the probability of the GS system being cost-effective at various willingness-to-pay thresholds compared to the SoC (Figure 3). According to the CEAC, at a threshold of GBP 20,000 per QALY gained, the GS system has a 60.9% probability of being cost-effective compared to the SoC.

## 4. Discussion

Our findings suggest that the GS system, compared to the SoC, meets the standard criteria for being a cost-effective use of resources within the UK healthcare setting. There are few studies that focus on the cost-effectiveness of rehabilitation following THA or TKA, and to the best of our knowledge, this is the first study to assess the cost-effectiveness of the GS system for these patients, limiting direct comparisons with the existing literature. However, our results align with other economic evaluations of various rehabilitation components for THA or TKA patients, indicating that the GS system is more effective and cost-saving for the healthcare system compared to the usual care [27,28,29].

The use of cost-effectiveness analysis (CEA) in healthcare decision-making is well-documented. By integrating cost data with clinical outcomes, the CEA provides a comprehensive assessment of an intervention’s value. In the context of post-operative rehabilitation, the CEA can help determine whether the additional costs associated with advanced technologies like GS are justified by the improvements in patient outcomes [30,31].

Overall, this study contributes to the growing body of evidence supporting the cost-effectiveness of using the GS system in clinical practice for rehabilitating patients undergoing THA or TKA. Sensitivity analyses, conducted to test the robustness of our assumptions regarding the input parameters, showed that the GS system remains the more cost-effective option, even when the input values are varied around the mean.

The primary strength of this study lies in its use of real-life parameters, including resource use, probabilities, and costs, which were obtained at the individual level from the GS RCT study. This enhances the reliability and validity of the model’s parameters. However, this study has notable limitations. The GS RCT had a small sample size, which prevented subgroup analyses due to insufficient statistical power. Additionally, this study lacked a follow-up protocol and data. Addressing these limitations in future RCTs would be beneficial.

In this study, the healthcare costs per QALY were evaluated over a 17-week period, corresponding to the duration of the GS RCT. Given that osteoarthritis (OA) is a chronic condition with long-term implications [32], this 17-week timeframe is relatively short for assessing the cost-effectiveness. Consequently, there is a need for extended long-term analyses to better understand the full impact. However, this cost-effectiveness analysis suggests that a physical activity program for knee OA patients may yield significant long-term clinical and economic benefits. Thus, the benefits observed in our study could be even more pronounced over a longer time horizon.

Clinical guidelines by the NICE in the UK recommend a multidisciplinary and integrated approach to the assessment and management of osteoarthritis [6]. The guidelines emphasise the importance of patient education and self-management to improve understanding of the condition and its treatment. However, they currently provide economic analyses only for pharmacological and conventional treatments. To address this gap, it is recommended that future guidelines include economic evaluations of interventions specifically aimed at the rehabilitation of patients undergoing total hip arthroplasty (THA) and total knee arthroplasty (TKA).

This study limited the analysis to the NHS perspective, following the NICE reference case [26]. However, it is important to recognise that a significant portion of the economic burden of osteoarthritis (OA) stems from indirect costs and productivity losses [4]. A societal perspective could, therefore, offer a more comprehensive view of the total impact on society. Some guidelines advocate for adopting a broader societal perspective in economic analyses due to the chronic nature of OA [33]. Incorporating this perspective into sensitivity analyses could provide a more accurate representation of the overall burden associated with OA.

## 5. Conclusions

Our economic evaluation results indicate that the GS system may be a dominant strategy (delivering lower costs and improving the quality-of-life outcomes) compared to the current SoC. Even under less favourable assumptions, where the parameters with the highest level of uncertainty were varied, the GS showed to be a cost-effective alternative, assuming a GBP 20,000 WTP threshold.

Thus, our cost-effectiveness analysis suggests that GS in patients undergoing THA and TKA with gait and mobility issues is also potentially cost-saving compared to the SoC, reducing costs by GBP 450.56 per patient. Policymakers and practitioners should consider the possible cost benefits of introducing the GS as an alternative to the current practice and support further studies to strengthen the generalisability of the current findings.

## Figures and Tables

**Table 1:** Self-managed rehabilitation model inputs.

	Parameter	Deterministic Value	Probabilistic Value	Distribution	Source
**Transition Probabilities**	Self-Managed Rehabilitation (SMR-SoC)	0.200	0.208	Beta	Expert Opinion
	Self-Managed Rehabilitation (SMR-Int)	0.000	0.000	Beta	Expert Opinion
	SMR Response Probability	0.400	0.394	Beta	Expert Opinion
	SMR No Response Probability	0.600	0.606	Beta	Expert Opinion
**Costs (GBP)**	Physiotherapy Cost	10.333	11.126	Gamma	[18]
	Follow-Up Cost	0.000	0.000	Gamma	[18]
	Administration Cost	1.553	1.009	Gamma	[18]
**Utilities**	QoL GIR Responder	0.272	0.211	Beta	[21]
	QoL GIR No Responder	0.256	0.202	Beta	[21]

**Table 2:** Group/individual rehabilitation model inputs.

	Parameter	Deterministic Value	Probabilistic Value	Distribution	Source
**Transition Probabilities**	Group/Individual Rehabilitation (GIR-Soc)	0.800	0.792	Beta	Experts’ Opinion
	Group/Individual Rehabilitation (GIR-Int)	0.000	0.000	Beta	Expert Opinion
	GIR Response	0.400	0.399	Beta	[25]
	GIR No Response	0.600	0.601	Beta	[25]
**Costs (GBP)**	Physiotherapy Cost	48.000	55.851	Gamma	[24]
	Secondary Care Costs	57.000	49.387	Gamma	[24]
	Follow-Up Cost	0.000	0.000	Gamma	[24]
	Administration Cost	2.329	2.366	Gamma	[24]
**Utilities**	QoL GIR Responder	0.272	0.201	Beta	[25]
	QoL GIR No Responder	0.256	0.342	Beta	[25]

**Table 3:** GaitSmart intervention model parameters.

	Parameter	Deterministic Value	Probabilistic Value	Distribution	Source
**Transition Probabilities**	GaitSmart Rehabilitation (GSR)	1.000	1.000	Beta	Assumption
	GSR Response	0.800	0.812	Beta	[25]
	GSR No Response	0.200	0.188	Beta	[25]
	Primary Care	0.000	0.000	Beta	[25]
**Costs (GBP)**	GaitSmart Intervention Cost Per Patient per Session	10	–	–	Manufacturer
	Secondary Care Costs Per Patient per Session	6.75	6.73	Gamma	[24]
	Follow-Up Cost	0.000	0.000	Gamma	[24]
	Number of Sessions	4	–		[25]
**Utilities**	QoL GSR Responder	0.285	0.314	Beta	[25]
	QoL GSR No Responder	0.249	0.205	Beta	[25]

**Table 4:** Cost-effectiveness analysis results comparing GS vs. SoC.

Interventions	Mean Cost—£	Incremental Cost—£	Mean (QALYs)	Incremental (QALYs)	ICER—£
**SoC**	517.56	-	0.26	-	-
**GS**	67.00	−450.56	0.28	0.02	Dominant

GS: GaitSmart, ICER: incremental cost-effectiveness ratio, QALY: quality-adjusted life years, SoC: standard of care.

## Data Availability

Dataset available on request from the authors.

## Supplementary Materials

The following supporting information can be downloaded at: https://www.mdpi.com/article/10.3390/geriatrics9050129/s1, Table S1: Probabilistic sensitivity results comparing the GS vs. SoC.
