# Supplementary material for: Cost-Effectiveness of GaitSmart and an Artificial Intelligence Solution for Rehabilitation of Patients Undergoing Total Hip Arthroplasty (THA) and Total Knee Arthroplasty (TKA) in Older Population in the United Kingdom
Source: Geriatrics (Basel). 2024 Oct 5;9(5):129. doi: 10.3390/geriatrics9050129 (PMC11508099; doi:10.3390/geriatrics9050129)
Supplement: Supplementary file 1 [file geriatrics-09-00129-s001.zip › geriatrics-3040369-supplementary.pdf]

Table S1. Probabilistic sensitivity results comparing GS versus SoC

**Interventions**	**Mean Cost - £ (95%CI)**	**Incremental Cost - £ (95%CI)**	**Mean (QALYs) (95%CI)**	**Incremental (QALYs) (95%CI)**	**ICER - £ (95%CI)**
**SoC**	518.72 (421.87 – 622.24)	-	0.26 (0.16 – 0.38)	-	-
**GS**	67.15 (50.53 - 92.25)	-451.57 (-354.48 – -558.42)	0.28 (0.14 – 0.45)	0.02 (-0.17 – 0.22)	Dominant

CI: confidence interval, GS: GaitSmart, ICER: incremental cost-effectiveness ration, QALY: quality adjusted life years, SoC: standard of care
